# A Dual-stage Deep Learning Framework for Breast Ultrasound Image Segmentation and Classification

**DOI:** 10.1007/s10916-025-02298-6

**Published:** 2025-11-18

**Authors:** Pierangela Bruno, Megan Macrì, Carmine Dodaro

**Affiliations:** Department of Mathematics and Computer Science, University of Calabria, Rende, 87036 CS Italy

**Keywords:** Deep learning, Classification, Segmentation, Breast cancer

## Abstract

Deep Learning methods have become a powerful tool in medical imaging, with great potential to improve diagnostic accuracy and support early disease detection. This is especially critical for breast cancer, one of the most common cancers among women, where early detection of abnormal tissue is crucial to improving survival rates. In this paper, we explore the application of Deep Learning techniques to segment and classify breast masses as malignant or benign using ultrasound images, aiming to support breast cancer diagnosis. We propose a modular dual-stage pipeline that first segments suspicious regions and then classifies them into benign or malignant categories. The framework is designed to flexibly integrate different backbone architectures, allowing adaptation to task- or dataset-specific requirements. Experimental results show that, within this pipeline, DeepLabV3+ with a ResNet34 encoder provided the most accurate segmentation, while lightweight classifiers such as MobileNetV3-Small and EfficientNet-B0 yielded the best classification performance. Moreover, an ablation study was conducted to tune parameters and determine their optimal configuration. Finally, our approach was tested on two breast ultrasound datasets, and the results show promising improvements in diagnostic accuracy, demonstrating the potential of our method to enhance early breast cancer detection.

## Introduction

Artificial Intelligence (AI) encompasses a range of algorithms designed to replicate human cognitive functions, with Machine Learning (ML) and Deep Learning (DL) being two prominent paradigms. ML enables systems to learn patterns from data and make predictions without explicit programming, significantly advancing domains such as finance, natural language processing, and medical diagnostics [2]. DL, a specialized subset of ML, leverages deep neural networks capable of learning hierarchical representations directly from raw data, proving particularly effective in applications like image analysis, speech recognition, and medical imaging [3].

Within DL, Convolutional Neural Networks (CNNs) have emerged as a key architecture for processing image data due to their ability to capture spatial hierarchies. CNNs have demonstrated strong performance in tasks such as image classification, object detection, and segmentation, with notable success in medical imaging for disease diagnosis, including cancer [4].

Breast cancer remains the most commonly diagnosed cancer among women worldwide and is a leading cause of cancer-related mortality. Early and accurate diagnosis is critical for improving patient outcomes. According to the World Health Organization, breast cancer accounts for approximately 25% of cancer diagnoses in women globally [5]. Ultrasound imaging plays a vital role in breast cancer screening, particularly for patients with dense breast tissue where mammography may fall short [6]. However, the interpretation of ultrasound images is challenging due to inter-observer variability and the subtle differences between benign and malignant lesions.

To address these challenges, we present a dual-stage modular deep learning framework that first segments breast masses and then classifies them as benign or malignant. The pipeline is designed to flexibly accommodate different architectures in each stage, making it adaptable to diverse datasets and clinical scenarios. Experimental results show that, within this framework, DeepLabV3+ with a ResNet34 encoder provided the most accurate segmentation, while lightweight classifiers such as MobileNetV3-Small and EfficientNet-B0 achieved superior classification performance. This dual-stage architecture aims to enhance both the accuracy of lesion localization and diagnostic classification, supporting a more reliable and automated breast cancer assessment pipeline.

Our approach is validated on two publicly available breast ultrasound (BUS) datasets containing both benign and malignant lesions: (i) the BUSI dataset from Baheya Hospital in Egypt [7], and (ii) the Breast-Lesions-USG dataset from the Cancer Imaging Archive [8]. To improve model robustness and mitigate overfitting, we incorporate data augmentation and regularization techniques such as dropout, fostering improved generalization across diverse clinical scenarios.

Experimental findings confirm that our dual-stage framework effectively enhances both lesion segmentation and classification accuracy, outperforming traditional single-step models and demonstrating strong generalization across diverse datasets.

## Related Work

In recent years, several studies employed DL techniques for both classification and segmentation tasks, addressing a broad range of challenges across various domains [9–11]. These methods have shown remarkable progress in image analysis, enabling more accurate classification and segmentation in medical imaging.

CNN architectures, such as U-Net and its variants, have been widely used to segment breast tumors in ultrasound images. For example, Biesok et al. [12] proposed a three-stage breast tumor segmentation method based on an autoencoder CNN. It introduces a novel use of fuzzy connectedness with distance-adapted affinity to create hybrid pseudo-color images as input. Post-processing with the Chan-Vese active contour model refines the segmentation, achieving a median Dice score of around 0.86 on a 993-image multi-source dataset. Jabeen et al. [13] combined CNN-based feature extraction to select and fuse the best features, which are then classified using ML algorithms to improve BUS lesion classification accuracy.

Motivated by the interdependence of detection and diagnosis, recent research has shifted toward integrated frameworks that tackle segmentation and classification jointly. Podda et al. [14] proposed a fully automated DL pipeline for the segmentation and classification of BUS images. It combines multiple CNN architectures into ensemble models and introduces a novel cyclic mutual optimization process that iteratively improves segmentation and classification results using each other’s output. Trained on the BUSI dataset, the authors evaluated their approach on both binary and multi-classification settings, achieving in the latter a classification accuracy of 91.14% and a Dice coefficient of 82.59%, outperforming individual models and showing competitive results compared to state-of-the-art methods.

Instead of relying on step-by-step pipelines, more advanced end-to-end multi-task learning frameworks have been developed to handle both segmentation and classification at the same time within a single network. These multi-task CNN architectures share features between tasks, allowing the model to learn from both pixel-level and image-level information. For instance, Lu et al. [15] presented a DL framework, MTL-OCA, designed for the simultaneous segmentation and classification of BUS images. The model leverages high-level features from segmentation masks to improve classification performance. Evaluated on public datasets, MTL-OCA outperforms several state-of-the-art methods in both tasks.

Another notable trend is the incorporation of advanced network architectures such as attention modules and Transformers into segmentation-classification pipelines. Umer et al. [16] introduced a dual-decoder U-shaped network with attention gates for segmentation alongside a parallel CNN classifier, forming a unified framework that achieved high accuracy in both tasks. More recently, Zhang et al. [17] introduced a multi-task deep learning network for breast ultrasound tumor segmentation and classification, combining CNN and Transformer streams to capture both local and global features. Tested on two datasets, their approach improved classification accuracy from 73.64% to 80.21% on an external validation set.

Building upon these advances, our approach introduces a dual-stage modular pipeline where segmentation and classification are explicitly decoupled but integrated in a unified workflow. Unlike multi-task models that share representations, our framework optimizes each stage independently and then fuses them, allowing for flexibility in selecting the most effective architectures. In our experiments, DeepLabV3+ with a ResNet34 encoder achieved the best segmentation accuracy, while MobileNetV3-Small and EfficientNet-B0 provided the most reliable classification results.

To address common issues like limited data and overfitting, we apply comprehensive data augmentation and pre-processing techniques. This not only improves the model’s robustness but also helps it generalize better to new cases. Our approach strikes a balance between performance and practicality, delivering strong results in both segmentation and classification while remaining efficient, making it well-suited for real-world clinical use.

## Proposed Approach

**Fig. 1:**
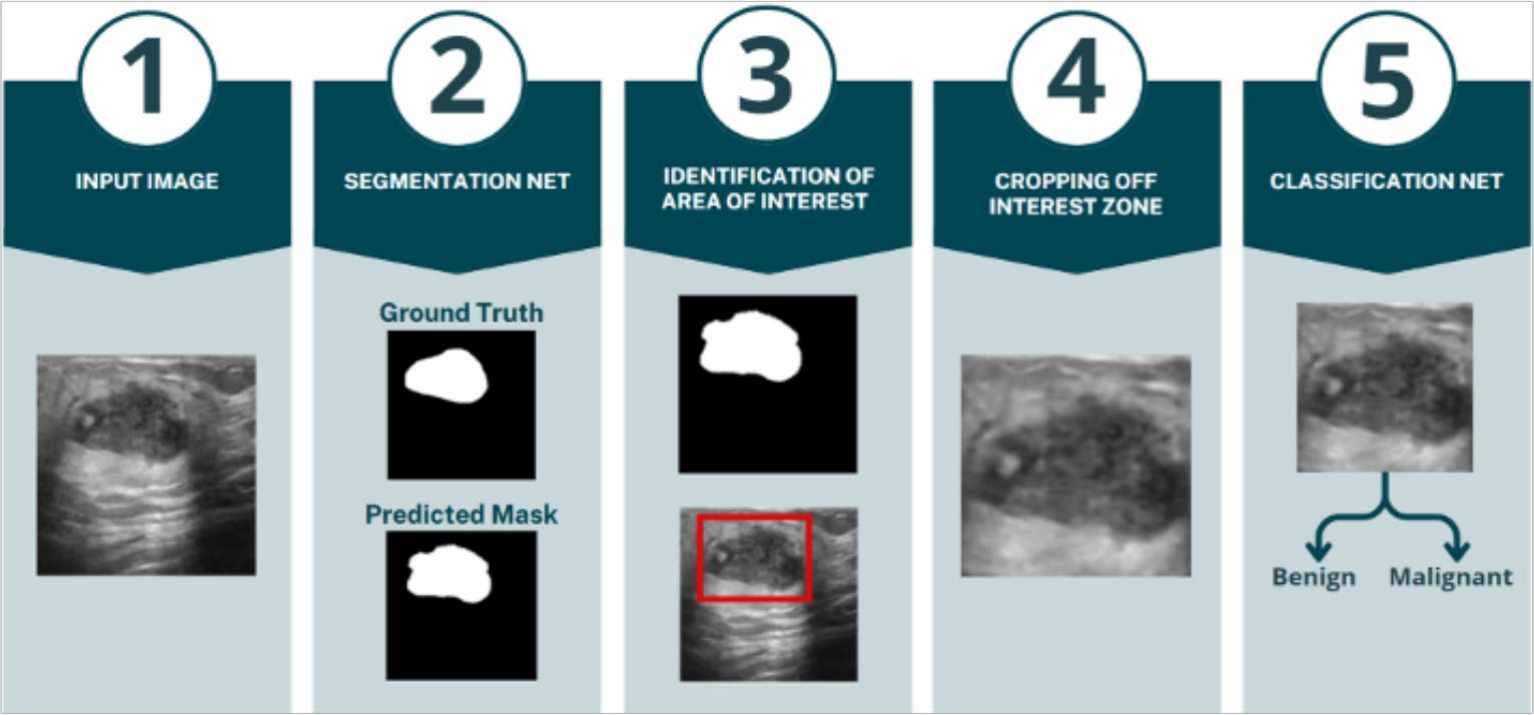
Workflow of the proposed approach

Our approach is composed of different steps, as shown in Fig. 1. After pre-processing and reading the images (Step 1), we perform breast mass segmentation using the U-Net architecture (Step 2). When the tumors are segmented, we isolate and extract the Region-of-Interest (ROI) corresponding to the predicted masses (Steps 3–4). Finally, we classify these segmented areas. In our approach, the segmenter is trained with a composite loss, producing clean and well-localized masks. The classifier then operates on these controlled crops using a pretrained backbone, with only the last layers partially unfrozen. This allows the model to adapt features with minimal changes, helping to avoid overfitting. Thanks to the modular design, our approach remains compact and easy to maintain: components can be swapped or upgraded independently, and widely used encoders/backbones with pretrained weights can be reused across datasets.

### Segmentation

We evaluated encoder–decoder architectures (U-Net, FPN and DeepLabV3+), each instantiated with either a ResNet-34 or EfficientNet-B0 encoder. In addition, we included a “traditional” U-Net since its well-established success in medical imaging applications [18, 19]. The network consists of four blocks in both the contracting path and the expansive path. In the contracting path, each block has two convolutional layers with ReLU activation and “same" padding, and each convolutional layer is composed of kernels with a size of 3x3. At the end of each block, there is a Max Pooling layer with a size of 2x2. After the layers of the encoder, there are two convolutional layers with 3x3 kernels and another transposed convolutional layer with 2x2 kernels that lead to the layers of the decoder. The blocks in the expansive path have two convolutional layers with 3x3 kernels and a transposed convolutional layer with 2x2 kernels. Training uses a composite objective that balances calibration and set overlap: a combination of binary cross-entropy (weight $0.4$) with the complements of Dice and IoU (each with weight $0.5$). From the binarized mask we derive a deterministic region of interest.

### Classification

Classification is applied solely on the cropped images. The image is replicated to three channels where required by the backbone. We use pretrained convolutional and transformer backbones (e.g., ResNet-18/50, DenseNet-121, EfficientNet-B0, ConvNeXt-Tiny, MobileNetV3-Small, ViT-B/16) followed by a lightweight prediction head. Two heads are considered: a linear layer and a shallow MLP with hidden sizes $(128,64)$ and optional dropout. To control the degree of transfer without altering the backbone topology, we freeze the backbone and optionally partially unfreeze only its last $K$ parameter groups (with $K\in \{0,10\}$ in our study). The classifier is trained with binary cross-entropy with logits.

## Dataset Description

**Fig. 2:**
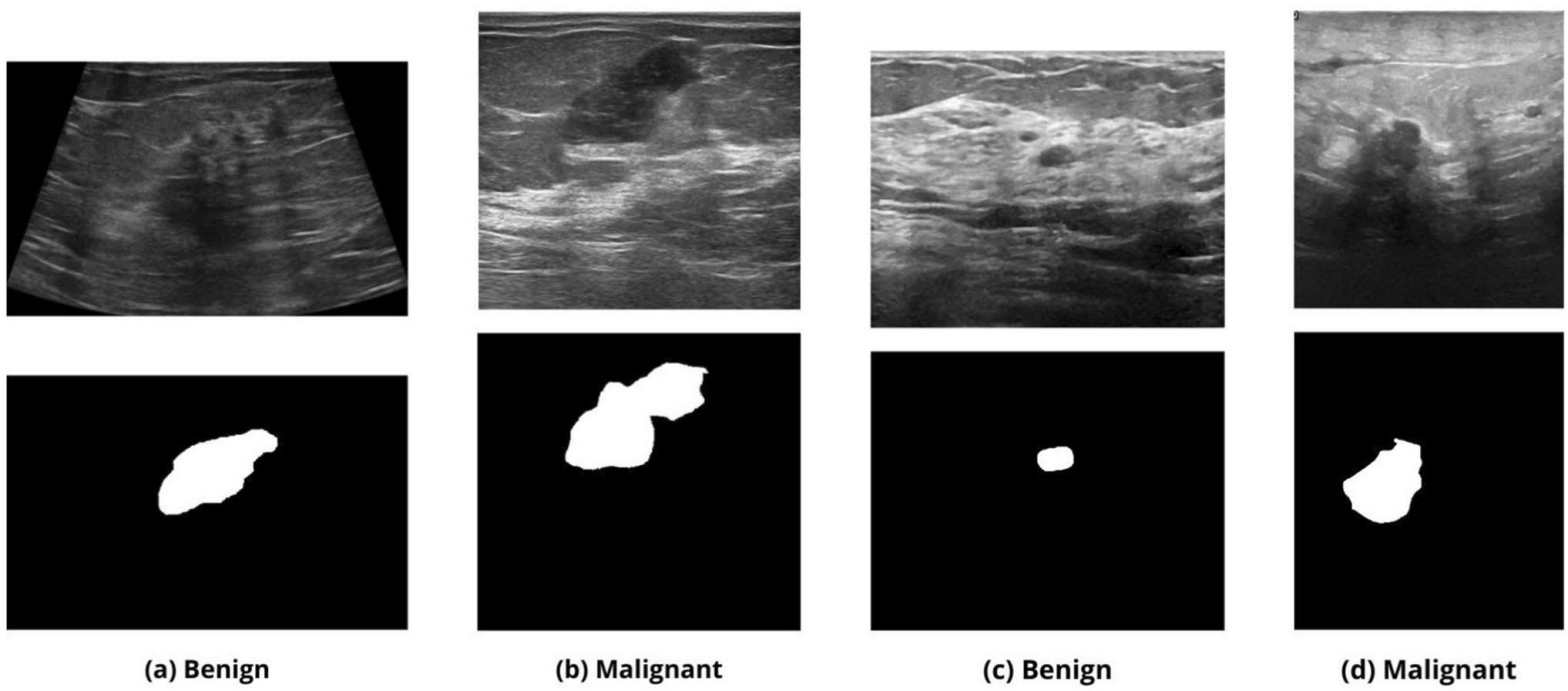
Example images from the BUSI (a-b) and Breast-Lesions-USG dataset (c-d). (a/c) Benign tumor with its corresponding segmentation (top to bottom). (b/d) Malignant tumor with its corresponding segmentation (top to bottom)

In our study, we use two publicly available breast ultrasound datasets:

1. **BUSI Dataset** [7] which comprises 780 ultrasound images from approximately 600 patients aged 25–75, collected at Baheya Hospital in Egypt. The images are divided into three categories: 487 benign, 210 malignant, and 83 normal (no lesion). Each image is provided with a corresponding binary segmentation mask. Images were converted from DICOM to PNG format at $500\times 500$ resolution, and subsequently resized to $256\times 256$ and converted to grayscale for processing. An example of images for each category is shown in Fig. 2 (a-b).
2. **Breast-Lesions-USG Dataset** [8] sourced from the Cancer Imaging Archive, which includes 256 patients with 266 annotated breast lesions (both benign and malignant), acquired using various ultrasound devices. The images, originally in PNG format and variable in size, were standardized to $256\times 256$ grayscale and include binary masks for each lesion. Variability in image quality reflects diverse clinical conditions. This dataset was used primarily for cross-dataset validation to test model generalization on data from a different distribution. An example of images for each category is shown in Fig. 2 (c-d).

To enhance model robustness and mitigate overfitting, various data augmentation techniques were applied including rotations, translations, zooming, and flipping. To prevent data leakage and ensure a fair evaluation, data augmentation was strictly applied only to the training set, after the dataset had been split at the patient level into training, validation, and test subsets. Augmentation was not applied to the validation or test sets, in order to preserve the integrity and objectivity of performance assessment. During preprocessing, masks were binarized and standardized to ensure consistency.

In our experiments, we focused only on the benign and malignant classes, yielding a refined subset of 697 images from the BUSI dataset.

## Experimental Tuning and Optimization

This section outlines the experimental design, dataset handling strategies, and optimization techniques employed to enhance segmentation and classification performance. The experiments were conducted under three scenarios:

1. **Scenario A:** Training, validation, and testing on the BUSI dataset.
2. **Scenario B:** Training, validation, and testing on both BUSI and Breast-Lesions-USG datasets.
3. **Scenario C:** Training and validation on BUSI; testing on Breast-Lesions-USG.

During the Step 2 of our workflow, we split the dataset into training, validation, and testing sets with a 50-15-35 ratio. Each patient’s images were confined to a single subset, preserving the integrity of model evaluation on unseen data.

Moreover, to minimize overlap between segmentation and classification data, and thereby reduce the exclusion of images from classification, we deliberately limited the size of the segmentation training set. This was essential to ensure that regions of interest identified during segmentation did not bias the classification phase.

To address class imbalance and mitigate overfitting, we employed oversampling to equalize class distributions and implemented extensive data augmentation; in particular, we applied conservative geometric augmentation (augmentation techniques included 20$^{\circ }$ rotations, 20% horizontal and vertical translations, zooming, and horizontal flipping) to preserve lesion morphology and enhance the model’s generalizability.

All experimental configurations involved thorough hyperparameter tuning, exploration of multiple network architectures, and optimization of loss functions. Segmentation performance was evaluated using accuracy, Intersection over Union (IoU), and Dice score, while classification was assessed using precision, recall, F1-score, accuracy, and Area Under the Curve (AUC). Statistical significance of performance differences was tested with the Wilcoxon signed-rank test, considering results significant at p < 0.05.

## Results and Discussion

### Scenario A

#### Segmentation

Table 1 lists the top segmentation configurations for Scenario A, ranked by Dice coefficient. The best-performing configuration was DeepLabV3+ with a ResNet34 encoder, initialized with 64 feature channels, achieving a Dice score of 0.769, IoU of 0.625, and pixel accuracy of 0.958. U-Net with a ResNet34 encoder followed closely (Dice 0.752), showing that residual encoders remain a robust choice even in the simpler U-Net design. When using 32 feature channels, the DeepLabV3+ model reached a Dice score of 0.747, which is slightly lower than the results of larger models. This shows that increasing model capacity can improve segmentation performance. Furthermore, our experiments show that replacing the ResNet34 backbone with EfficientNet-B0 leads to a moderate decrease in segmentation accuracy, with both U-Net and DeepLabV3+ reaching lower Dice (0.734) and IoU (0.580) scores. Importantly, the best-performing models shows a high pixel accuracy (0.958), which indicates that the approach is able to properly identify boundary localization.

For training, we optimized segmentation networks using the Adam optimizer with a learning rate of $1\times 10^{-4}$. We employed a composite loss function combining Binary Cross-Entropy, Dice loss, and IoU loss, to balance pixel-wise fidelity with overlap-based region metrics. A ReduceLROnPlateau scheduler (factor 0.1, patience 3) was applied to adapt the learning rate, and early stopping with a patience of 10 epochs was used to prevent overfitting.

**Table 1:** Top segmentation results for Scenario A, ranked by Dice

Architectures	Encoder	Base Channel	Dice	IoU	Acc.
DeepLabV3+	ResNet34	64	**0.769**	**0.625**	**0.958**
U-Net	ResNet34	32	0.752	0.603	0.956
DeepLabV3+	ResNet34	32	0.747	0.596	0.954
U-Net	EfficientNet-B0	32	0.734	0.580	0.953
DeepLabV3+	EfficientNet-B0	32	0.734	0.580	0.954

Best values in bold

**Fig. 3:**
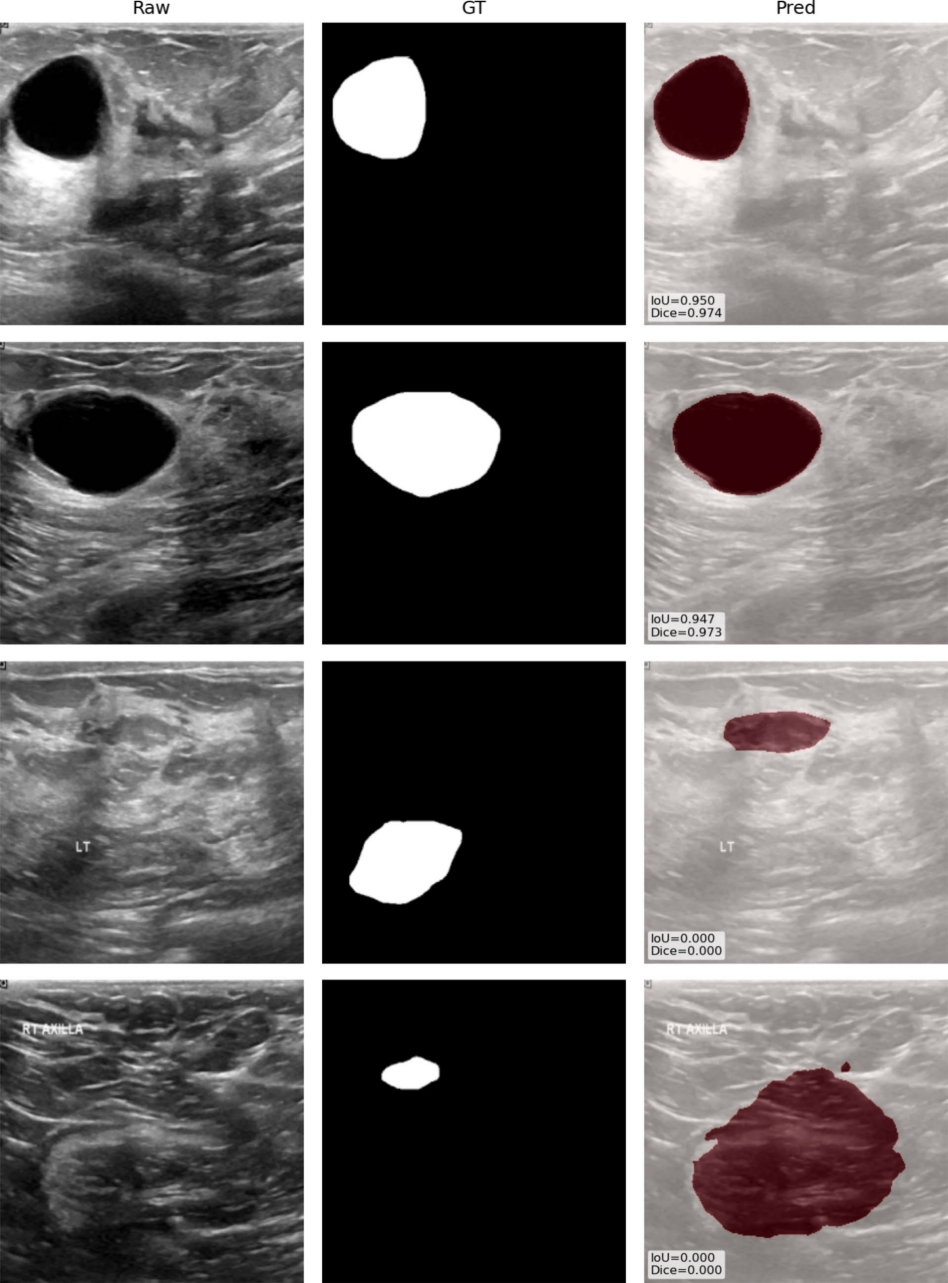
Example of best (first two rows) and worst (last two rows) cases in Scenario A along raw images and ground truth masks. IoU and Dice results are also reported

A visual example of the results is shown in Fig. 3.

#### Classification

After identifying the best segmentation model (DeepLabV3+ with ResNet34, 64 feature channels), we evaluated multiple classification backbones on the ROI masks. Table 2 reports the top-performing classifiers, ranked by AUC, and includes not only the backbone and head design but also the fine-tuning parameters such as dropout settings and unfreeze k layers, which indicates the number of last backbone layers unfrozen for fine-tuning, while the remaining layers stay frozen. The best result is achieved using MobileNetV3-Small with an MLP head, with 10 unfreeze layers and dropout set at 0.5. This configuration achieved an AUC of 0.990, F1 of 0.944, precision of 0.966, and recall of 0.924. EfficientNet-B0 also reached the top AUC (0.990), with higher recall (0.947) but slightly lower precision. DenseNet121 also performed strongly (AUC of 0.988), while ResNet50, despite its depth, did not surpass lighter models. ResNet18 reached the same AUC (0.988) but with a drop in F1 score (0.911).

Classification networks were trained using the Adam optimizer with a learning rate of $1\times 10^{-3}$. The loss function was Binary Cross-Entropy with logits (BCEWithLogitsLoss). Similarly to segmentation, a ReduceLROnPlateau scheduler (factor 0.1, patience 3) was employed, and early stopping (patience 10 epochs) was adopted to select the best-performing fold model.

**Table 2:** Top classification results on ROIs, ranked by AUC

Backbone	Head	unfreeze k layers	Dropout	AUC	F1	Precision	Recall
MobileNetV3-Small	MLP (128,64)	10	0.5	**0.990**	**0.944**	**0.966**	0.924
EfficientNet-B0	Linear	10	0.0	**0.990**	0.941	0.935	**0.947**
ResNet50	Linear	10	0.0	0.988	0.934	0.944	0.924
DenseNet121	MLP (128,64)	10	0.5	0.988	0.945	0.956	0.935
ResNet18	MLP (128,64)	10	0.5	0.988	0.911	0.932	0.891

Best values in bold

### Scenario B

#### Segmentation

Building upon the best architecture and hyperparameter configuration identified in Scenario A, we conducted a series of experiments to further explore the impact of various training strategies and the integration of a new unseen dataset. In particular, we wanted to test models’ capabilities in segmenting and classifying images from different sources. The results confirm the effectiveness of the design choices from Scenario A as well as their robustness across datasets. Indeed, in the segmentation task, the approach achieved an IoU of 0.650, a Dice coefficient of 0.788, and a pixel accuracy of 0.964. In particular, the consistency of Dice and IoU values across datasets demonstrates that the architectures identified in Scenario A extend reliably to the new ultrasound source.

#### Classification

For classification, we reused the best-performing architecture from Scenario A. The model achieved a recall of 0.927, precision of 0.927, F1 score of 0.927, and an AUC of 0.972, demonstrating strong generalization performance and validating the robustness of the fine-tuning strategy established earlier. Indeed, the balanced precision and recall demonstrate that the classifier is both sensitive to malignant lesions and robust against false positives.

### Scenario C

#### Segmentation

To further investigate the generalization ability of the proposed models, we conducted an experiment where both training and validation were performed solely on the BUSI dataset, as done in Scenario A, while inference was carried out exclusively on the Breast-Lesions-USG dataset. This setup was intended to evaluate the robustness of the models when applied to previously unseen data drawn from a different distribution.

**Fig. 4:**
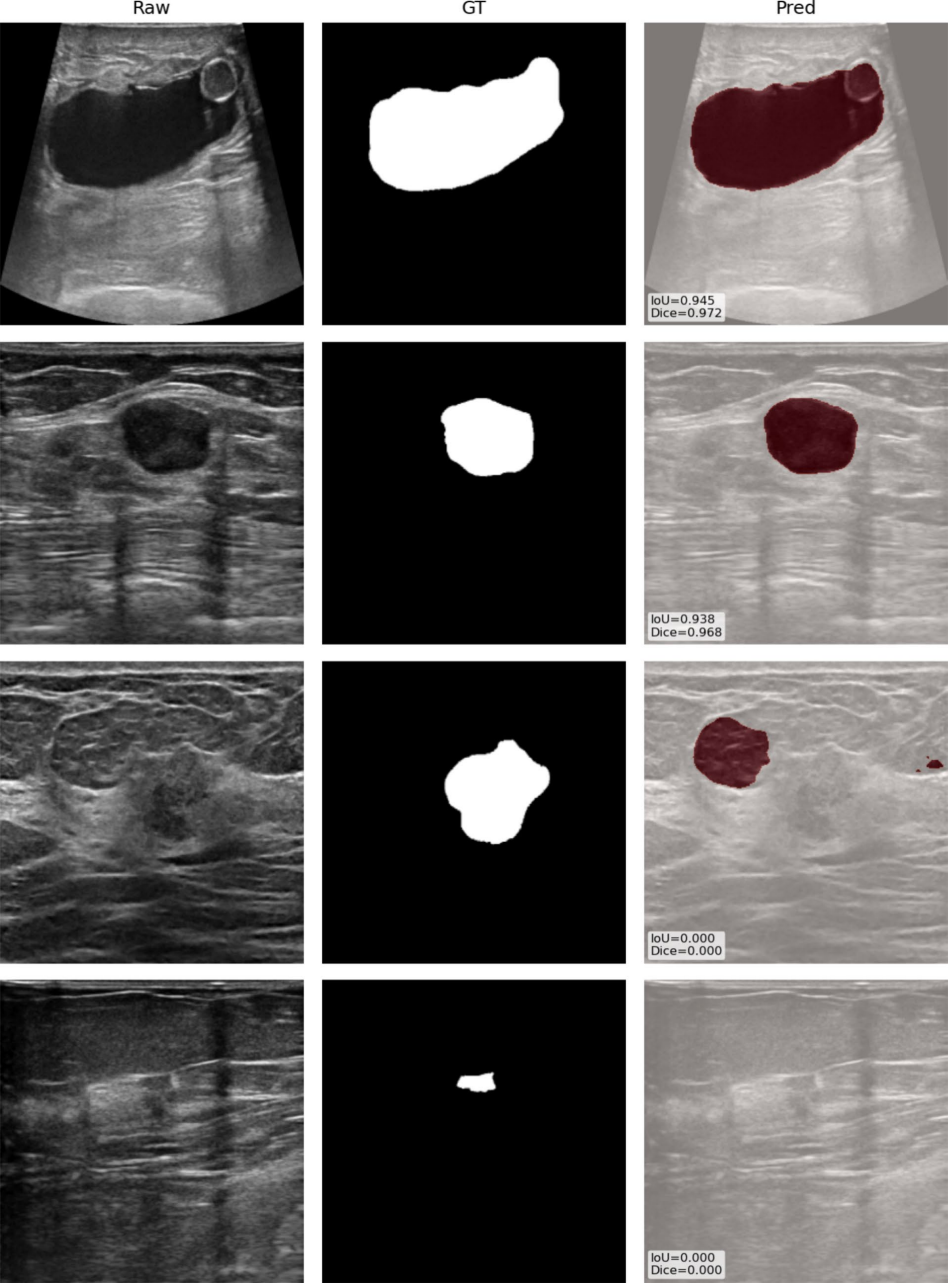
Example of best (first two rows) and worst (last two rows) cases in Scenario C along raw images and ground truth masks. IoU and DICE results are also reported

Using the best-performing segmentation configuration identified in Scenario A, we assessed the model’s performance on the USG dataset. The model demonstrated strong generalization, achieving an accuracy of 0.962. The Dice coefficient and IoU score were 0.738 and 0.602, respectively, supporting the model’s ability to generalize its spatial predictions across datasets, despite a slight drop in performance compared to previous scenarios. A visual example of the best and worst results is shown in Fig. 4.

#### Classification

Similar to the segmentation experiments, we used the model and optimal configuration from Scenario A to classify the USG dataset. It achieved an F1 score of 0.795, with recall and precision of 0.761 and 0.833, respectively. The AUC reached 0.876, confirming good discriminatory ability even under cross-dataset evaluation. While classification performance decreased compared to Scenario A, such a reduction is expected due to differences in image quality, acquisition protocols, and lesion appearance between datasets. Importantly, the model still preserved a strong ability to separate benign from malignant cases, as reflected by the balanced precision–recall values and the solid AUC score.

**Table 3:** Quantitative comparison between our proposed approach, Mask R-CNN, and Multi-task Transformers across segmentation and classification tasks. Best values in bold

Method	Scenario	Segmentation			Classification				
		Acc.	IoU	Dice	Acc.	Prec.	Rec.	F1	AUC
Our Approach	A	**0.958**	0.625	**0.769**	**0.952**	**0.966**	**0.924**	**0.944**	**0.990**
	B	0.964	0.650	**0.788**	**0.947**	**0.927**	**0.927**	**0.927**	**0.972**
	C	**0.962**	**0.602**	**0.738**	**0.802**	**0.833**	**0.761**	**0.795**	**0.876**
Mask R-CNN	A	0.957	**0.691**	0.762	0.809	0.768	0.744	0.754	0.744
	B	**0.969**	**0.657**	0.748	0.784	0.769	0.775	0.772	0.775
	C	0.937	0.449	0.523	0.655	0.457	0.430	0.443	0.668
Multi-task Transformers	A	0.953	0.512	0.617	0.898	0.944	0.654	0.773	0.923
	B	0.947	0.426	0.540	0.794	0.675	0.643	0.659	0.868
	C	0.960	0.450	0.560	0.690	0.600	0.612	0.606	0.753

## Comparison to Single-step Model

To demonstrate the effectiveness of our approach, we compared our results against Mask R-CNN [20], a well-established model for instance segmentation. Built on top of Faster R-CNN, it introduces an additional branch for predicting segmentation masks, enabling precise pixel-level delineation of objects alongside standard object detection. This makes it particularly suitable for medical imaging applications, where accurate lesion localization is critical. For our experiments, we used a ResNet-50 backbone with a Feature Pyramid Network (FPN), initialized with pretrained weights from the COCO dataset. The model was fine-tuned to segment breast lesions and classify them as either benign or malignant. After evaluating multiple configurations, the best results were obtained by training for 30 epochs using Stochastic Gradient Descent (SGD) with a learning rate of 0.001, momentum of 0.9, and weight decay of 0.0005.

The quantitative results in Table 3 clearly highlight the advantages of our approach over Mask R-CNN across both segmentation and classification tasks. While Mask R-CNN achieves reasonable segmentation, particularly in Scenarios A and B with IoU scores of 0.691 and 0.657, our method shows a more balanced performance across all metrics, maintaining competitive segmentation accuracy (up to 0.964 in Scenario B) and superior Dice scores (0.769, 0.788, and 0.738 across Scenarios A,B and C). Notably, in the cross-domain evaluation (Scenario C), our framework outperforms Mask R-CNN with higher IoU (0.602 vs. 0.449) and Dice (0.738 vs. 0.523), while also retaining a strong pixel accuracy of 0.962.

In terms of classification, our approach demonstrates a clear advantage. In Scenarios A and B, it achieves accuracies of 0.952 and 0.947 with F1-scores of 0.944 and 0.927, markedly higher than Mask R-CNN’s best classification accuracy of 0.809 and F1-score of 0.772. Even in the challenging cross-domain setting (Scenario C), our method maintains robust discrimination with an AUC of 0.876 and balanced precision–recall trade-offs (precision 0.833, recall 0.761, F1 0.795). In contrast, Mask R-CNN shows larger declines in classification metrics under domain shift, confirming the stronger generalization of our dual-stage pipeline. Overall, these findings underscore the superiority of our approach in delivering both accurate lesion localization and reliable diagnostic classification, with greater robustness and generalizability compared to single-step and multi-task alternatives.

Furthermore, when compared with existing methods evaluated under similar conditions (Scenario A), such as the approach by Podda et al. [14], our approach shows improvements. As discussed in Section Related Work, the authors evaluated their method on both binary and multi-class classification tasks; however, they did not report binary classification results specifically on the BUSI dataset. Therefore, we limited our comparison to the segmentation task, where their approach achieved an IoU of 0.659 and a Dice coefficient of 0.725. In contrast, our method, in Scenario A, obtained an IoU of 0.625 and a Dice coefficient of 0.769. While our IoU is slightly lower, the higher Dice score indicates a more balanced segmentation with improved overall region coverage, despite a marginal reduction in exact overlap.

## Multi-task Swin Transformer Baseline

To further strengthen our evaluation and align with recent advances, we implemented a multi-task baseline that jointly optimizes segmentation and classification with a shared Swin Transformer encoder [21]. Specifically, the model uses a pretrained Swin backbone (patch size 4, window size 7) as shared feature extractor, a lightweight decoder with two upsampling and convolutional blocks for lesion mask prediction, and an image-level classification head with two fully connected layers (ReLU and dropout 0.3) operating on the pooled encoder representation. Inputs are resized to $256\times 256$ RGB, and training follows a composite loss, combining BCEWithLogits for segmentation and cross-entropy for classification. Optimization uses AdamW (learning rate $10^{-4}$, weight decay $10^{-4}$). As summarized in Table 3, the multi-task Swin baseline achieves competitive results in the in-domain setting (Scenario A), reaching classification accuracy of 0.898 and an AUC of 0.923, with segmentation Dice of 0.617. When trained on heterogeneous sources (Scenario B), it maintains acceptable performance (Dice 0.540, classification accuracy 0.794, AUC 0.868), confirming that shared representations can generalize to mixed datasets. However, in the most challenging cross-domain evaluation (Scenario C), the model shows a more evident decline in classification (accuracy 0.690, F1-score 0.606, AUC 0.753), although segmentation remains stable (Dice 0.560, pixel accuracy 0.960). These findings highlight a trade-off: multi-task learning with Transformers is efficient and achieves strong in-domain performance, but several studies have shown that these approaches are sensitive to distribution shifts and often degrade when tested outside their training domain [22, 23]. In contrast, our dual-stage pipeline shows greater robustness under domain shift and offers modularity, which is advantageous in clinical workflows where segmentation and classification components may need to be validated or fine-tuned independently.

## Conclusion

We presented a dual-stage modular pipeline for BUS image analysis, where segmentation and classification are explicitly decoupled but integrated in a single workflow. This design enables components to be swapped or upgraded independently, offering flexibility and adaptability to different clinical scenarios. Across experiments, DeepLabV3+ with a ResNet34 encoder achieved the best segmentation results, while MobileNetV3-Small and EfficientNet-B0 provided superior classification accuracy.

The method was tested across different datasets and scenarios, showing strong performance in both segmentation and classification tasks. Compared to single-step approaches such as Mask R-CNN and to multi-task Transformer-based methods (including Swin Transformer baselines), our pipeline consistently achieved better and more robust results, especially in terms of generalization across datasets.

While segmentation performance remained high across datasets, classification was more affected by domain differences, suggesting the need for future work on domain adaptation. Overall, these results highlight the potential of the proposed framework to support more accurate and automated breast cancer diagnosis. Next steps will focus on improving generalizability to further enhance clinical applicability.

## Data Availability

No datasets were generated or analysed during the current study.

## References

[1] Macrì, M., Bruno, P., and Dodaro, C., Deep learning approaches for segmentation and classification of breast ultrasound images. Proceedings of the 3rd Workshop on Artificial Intelligence for Healthcare (HC@AIxIA 2024). CEUR Workshop Proceedings,vol. 3880, pp. 224–232, 2024.

[2] Jordan, M. I., and Mitchell, T. M., Machine learning: Trends, perspectives, and prospects. *Science* 349(6245):255–260, 2015. 26185243 10.1126/science.aaa8415

[3] LeCun, Y., Bengio, Y., and Hinton, G., Deep learning. *Nature* 521(7553):436–444, 2015. 26017442 10.1038/nature14539

[4] Aggarwal, R., Sounderajah, V., Martin, G., Ting, D. S., Karthikesalingam, A., King, D., Ashrafian, H., and Darzi, A., Diagnostic accuracy of deep learning in medical imaging: A systematic review and meta-analysis. *NPJ Digit. Med.* 4(1):65, 2021. 10.1038/s41746-021-00438-z PMC8027892 33828217

[5] Wilkinson, L., and Gathani, T., Understanding breast cancer as a global health concern. *Br. J. Radiol.* 95(1130):20211033, 2022. 10.1259/bjr.20211033 PMC8822551 34905391

[6] Guo, R., Lu, G., Qin, B., and Fei, B., Ultrasound imaging technologies for breast cancer detection and management: A review. *Ultrasound Med. Biol.* 44(1):37–70, 2018. 10.1016/j.ultrasmedbio.2017.09.012 PMC6169997 29107353

[7] Al-Dhabyani, W., Gomaa, H. K. M., and Fahmy, A., *Dataset of breast ultrasound images*. Elsevier, vol. 28, no. 104863, 2020. 10.1016/j.dib.2019.104863 PMC6906728 31867417

[8] Pawłowska, A., Ćwierz-Pieńkowska, A., Domalik, A., Jaguś, D., Kasprzak, P., Matkowski, R., Fura, Ł., Nowicki, A., and Żołek, N., Curated benchmark dataset for ultrasound based breast lesion analysis. *Sci. Data* 11(1):148, 2024. 10.1038/s41597-024-02984-z PMC10830496 38297002

[9] Roß, T., Bruno, P., Reinke, A., Wiesenfarth, M., Koeppel, L., Full, P. M., Pekdemir, B., Godau, P., Trofimova, D., Isensee, F. et al., Beyond rankings: Learning (more) from algorithm validation. *Med. Image Anal.* 86:102765, 2023. 10.1016/j.media.2023.102765 36965252

[10] Bruno, P., Spadea, M. F., Scaramuzzino, S., De Rosa, S., Indolfi, C., Gargiulo, G., Giugliano, G., Esposito, G., Calimeri, F., and Zaffino, P., Assessing vascular complexity of paod patients by deep learning-based segmentation and fractal dimension. *Neural Comput. Appl.* 34(24):22015–22022, 2022.

[11] Jiang, H., Diao, Z., Shi, T., Zhou, Y., Wang, F., Hu, W., Zhu, X., Luo, S., Tong, G., and Yao, Y.-D., A review of deep learning-based multiple-lesion recognition from medical images: Classification, detection and segmentation. *Comput. Biol. Med.* 157:106726, 2023. 10.1016/j.compbiomed.2023.106726 36924732

[12] Biesok, M., Juszczyk, J., and Badura, P., Breast tumor segmentation in ultrasound using distance-adapted fuzzy connectedness, convolutional neural network, and active contour. *Sci. Rep.* 14(1):25859, 2024. 10.1038/s41598-024-76308-x PMC11519628 39468220

[13] Jabeen, K., Khan, M. A., Alhaisoni, M., Tariq, U., Zhang, Y. -D., Hamza, A., Mickus, A., and Damaševičius, R., Breast cancer classification from ultrasound images using probability-based optimal deep learning feature fusion. *Sensors* 22(3):807, 2022. 10.3390/s22030807 10.3390/s22030807 PMC8840464 35161552

[14] Podda, A. S., Balia, R., Barra, S., Carta, S., Fenu, G., and Piano, L., Fully-automated deep learning pipeline for segmentation and classification of breast ultrasound images. *J. Comput. Sci.* 63:101816, 2022.

[15] Lu, Y., Sun, F., Wang, H., and Yu, K., Automatic joint segmentation and classification of breast ultrasound images via multi-task learning with object contextual attention. *Front. Oncol.* 15:1567577, 2025. 10.3389/fonc.2025.1567577 PMC12011763 40265029

[16] Umer, M. J., Sharif, M., and Wang, S. -H., Breast cancer classification and segmentation framework using multiscale cnn and u-shaped dual decoded attention network. *Expert Syst.* 13192, 2022.

[17] Zhang, Y., Zeng, B., Li, J., Zheng, Y., and Chen, X., A multi-task transformer with local-global feature interaction and multiple tumoral region guidance for breast cancer diagnosis. *IEEE J. Biomed. Health Inform.* 28(11):6840–6853, 2024. 10.1109/JBHI.2024.3454000 39226204

[18] Ronneberger, O., Fischer, P., and Brox, T., U-net: Convolutional networks for biomedical image segmentation. In: *Medical Image Computing and Computer-assisted intervention–MICCAI 2015: 18th International Conference*, Munich, Germany, October 5-9, 2015, Proceedings, Part III 18, pp. 234–241, 2015. Springer.

[19] Siddique, N., Paheding, S., Elkin, C. P., and Devabhaktuni, V., U-net and its variants for medical image segmentation: A review of theory and applications. *IEEE Access* 9:82031–82057, 2021.

[20] He, K., Gkioxari, G., Dollár, P., and Girshick, R., Mask r-cnn. In: *Proceedings of the IEEE international conference on computer vision*, pp. 2961–2969, 2017.

[21] Liu, Z., Lin, Y., Cao, Y., Hu, H., Wei, Y., Zhang, Z., Lin, S., and Guo, B., Swin transformer: Hierarchical vision transformer using shifted windows. In: *Proceedings of the IEEE/CVF international conference on computer vision*, pp. 10012–10022, 2021.

[22] Zhang, C., Zhang, M., Zhang, S., Jin, D., Zhou, Q., Cai, Z., Zhao, H., Liu, X., and Liu, Z., Delving deep into the generalization of vision transformers under distribution shifts. In: *Proceedings of the IEEE/CVF conference on computer vision and pattern recognition*, pp. 7277–7286, 2022.

[23] Alijani, S., Fayyad, J., and Najjaran, H., Vision transformers in domain adaptation and domain generalization: A study of robustness. *Neural Comput. Appl.* 36(29):17979–18007, 2024.

